# Effects of exercise and diet interventions on obesity-related sleep disorders in men: study protocol for a randomized controlled trial

**DOI:** 10.1186/1745-6215-14-235

**Published:** 2013-07-26

**Authors:** Xiao Tan, Antti Saarinen, Tuija M Mikkola, Jarkko Tenhunen, Samu Martinmäki, Aki Rahikainen, Shumei Cheng, Niklas Eklund, Satu Pekkala, Petri Wiklund, Eveliina Munukka, Xinfei Wen, Fengyu Cong, Xi Wang, Yajun Zhang, Ina Tarkka, Yining Sun, Markku Partinen, Markku Alen, Sulin Cheng

**Affiliations:** 1 Department of Health Sciences, University of Jyväskylä, Rautpohjankatu 8, PO Box 35, 40700 Jyväskylä, Finland; 2 Central Finland Central Hospital, Central Finland Health Care District, Keskussairaalantie 19, 40620 Jyväskylä, Finland; 3 Helsinki Sleep Clinic, Vitalmed Research Center, Sitratori 3, Third floor, 00420 Helsinki, Finland; 4 Department of Mathematical Information Technology, University of Jyväskylä, (Agora), PO Box 35, 40014 Jyväskylä, Finland; 5 Institute of Intelligent Machines, Chinese Academy of Sciences, 350 Shushan Road, Hefei 230031, Anhui, People’s Republic of China; 6 Department of Medical Rehabilitation, Oulu University Hospital, Oulu, Finland; 7 Institute of Health Sciences, University of Oulu, Kajaanintie 50, Oulu 90220, Finland

**Keywords:** Lifestyle intervention, Sleep disorders, Quality of sleep, Obstructive sleep apnea, Insomnia, Sleep measurement, Obesity, Gut microbiota, Neurotransmitters

## Abstract

**Background:**

Sleep is essential for normal and healthy living. Lack of good quality sleep affects physical, mental and emotional functions. Currently, the treatments of obesity-related sleep disorders focus more on suppressing sleep-related symptoms pharmaceutically and are often accompanied by side effects. Thus, there is urgent need for alternative ways to combat chronic sleep disorders. This study will investigate underlying mechanisms of the effects of exercise and diet intervention on obesity-related sleep disorders, the role of gut microbiota in relation to poor quality of sleep and day-time sleepiness, as well as the levels of hormones responsible for sleep-wake cycle regulation.

**Methods/design:**

Participants consist of 330 (target sample) Finnish men aged 30 to 65 years. Among them, we attempt to randomize 180 (target sample) with sleep disorders into exercise and diet intervention. After screening and physician examination, 101 men with sleep disorders are included and are randomly assigned into three groups: exercise (n = 33), diet (n = 35), and control (n = 33). In addition, we attempt to recruit a target number of 150 healthy men without sleep disorders as the reference group. The exercise group undergoes a six-month individualized progressive aerobic exercise program based on initial fitness level. The diet group follows a six month specific individualized diet program. The control group and reference group are asked to maintain their normal activity and diet during intervention. Measurements are taken before and after the intervention. Primary outcomes include objective sleep measurements by polysomnography and a home-based non-contact sleep monitoring system, and subjective sleep evaluation by questionnaires. Secondary outcome measures include anthropometry, body composition, fitness, sleep disorder-related lifestyle risk factors, composition of gut microbiota and adipose tissue metabolism, as well as specific hormone and neurotranmitter levels and inflammatory biomarkers from venous blood samples.

**Discussion:**

It is expected that the improvement of sleep quality after exercise and diet intervention will be evident both in subjective and objective measures of quality of sleep. Additionally, the change of sleep quality induced by exercise and diet intervention is expected to be related to the changes in specific hormones and inflammatory biomarkers, and in the composition of gut microbiota.

**Trial registration:**

Current Controlled Trials ISRCTN77172005

## Background

We spend one-third of our lifetime in sleep. Sleep is absolutely essential for maintaining healthy physical, mental and emotional functions. Sleep disorder impairs one’s ability to think quickly, to work efficiently, and to associate freely, thus making one feel generally ’disconnected’ from the world. In some serious cases, the sleep disorder-related conditions may lead to serious neurasthenia and depression [1,2]. Obesity is a major risk factor for sleep disorders, which may cause symptoms such as obstructive sleep apnea (OSA) [3,4]. OSA has an estimated prevalence of 2% to 4% among adults aged 30 to 60 years and the proportion is increasing [5,6]. OSA is associated with significant morbidity and mortality due to accidents, cardiovascular diseases, and stroke [7,8]. In addition, insomnia is a common sleep disorder that impairs quality of life from both physiological and psychological aspects, with prevalence range from 10% to 40% in Western countries [9-11]. Besides insomnia, a considerable proportion of the general adult population reports chronic mild to moderate sleep complaints which result in long-term poor quality of sleep, as well as increased health care visits [12].

Two thirds of middle-aged sleep apneic men are obese, and visceral obesity has been observed to be a primary risk factor for OSA [7]. Obesity reflected by body weight and body mass index (BMI) is also related to poor subjective quality of sleep [13] and short sleep duration [14-18] among adults. A widely supported theory that obesity induces inflammatory reactions may explain the association between obesity and sleep disorders [19,20]. Elevated levels of inflammatory biomarkers, such as C-reactive protein (CRP) [21,22], interleukin-6 (IL-6) [22,23], and tumor necrosis factor-alpha (TNF-alpha) [24] are reported among OSA patients. People with non-apneic sleep disorders also demonstrate changed levels of inflammatory biomarkers [25-27]. Both short or excessive habitual sleep duration are linked with greater levels of CRP [25-27], while short sleep duration recoded by polysomnography (PSG) is related to higher levels of TNF-alpha [25]. However, it is still unclear whether inflammation causes sleep disorders or vice versa [28-31].

Sleep disorders elevate the risk of obesity by affecting eating patterns and other lifestyle factors. Short sleep duration is associated with higher energy intake, mostly due to increased consumption of saturated fat [32]. Weight loss from three months exercise training or one year diet control alleviates the symptom of OSA by lowering the apnea-hypopnea index (AHI) [33-35]. The possible association between weight loss and subjective quality and quantity of sleep is exhibited in some adult women [36]. Despite the change of body weight and BMI, lifestyle intervention through aerobic or resistance exercise may improve quality of sleep in middle-aged and older adults with sleep complaints [9,12]. However, more clinical trials are needed since very few previous studies have utilized objective sleep measurements [32,37].

Gut microbiota is suggested to act as an important factor regulating adipose metabolism, and affecting neurological functions. Animal studies show that gut microbiota affects energy harvest from diet and energy storage in the host. The association is reflected by lower metabolic rate, increased hepatic production of triglycerides, and promoted storage of triglycerides in adipocytes among mice with gut microbiota compared with their germ-free counterparts [38]. In human studies, composition of gut microbiota shows differences between obese and lean subjects [39], and certain types of gut microbiota may independently link to obesity-related metabolic disorder [40]. Moreover, gut microbiota may be involved in modulation of both central and peripheral nerve function, such as the hypothalamus-pituitary-adrenal (HPA) axis and thus associate with neuropsychiatric conditions including anxiety, depression, and sleep disorders [41,42]. Composition of gut microbiota can be altered through long-term dietary intervention [43]; however whether changing the human intestinal microbiota through lifestyle intervention helps to mediate sleep disorders has yet to be studied.

### Methods/design

The study aims to involve 330 Finnish men aged 30 to 65 years with or without sleep disorders. Participants are recruited from City of Jyväskylä health care centers in the Central Finland Health Care District and its surroundings via doctors’ referral and advertising in news media and on the internet between 28 April 2011 and 2 April 2013. Among the total 330 target number of participants, 180 are sleep disordered with OSA or insomnia (OSA is diagnosed and referred by a sleep specialist physician at the Central Hospital of Central Finland prior to the baseline assessments. Insomnia is reported first by the participants then examined by a physician prior to other baseline assessments. The definition of insomnia is based on the following symptoms: recurrent difficulty in falling asleep, too short sleep duration or poor quality of sleep during the previous three months [44]). The remaining 150 participants are healthy men without sleep disorders.

Among the 180 men with sleep disorders, 171 responded to the initial advertisement (response rate: 95%). A screening interview was carried out for interested participants who contacted the researchers. The screening covered the participant’s health and medical conditions and certain lifestyle factors such as physical activity, type of employment, and so on. Sixty-six participants did not meet the inclusion criteria. The remaining 105 participants were invited to the laboratory and evaluated by a physician to make sure that they could be included in the intervention study. The examination by a physician included cardiac and musculoskeletal status evaluations, risk evaluation concerning exercise tolerance [45], and family background-related diseases, through which four participants were excluded. Thereafter, baseline tests were performed. Participants with sleep disorders were randomized into three groups: exercise, diet, and control. The exercise and diet groups followed a six-month guided individualized exercise and diet counseling intervention program respectively, while the control group and reference group members were asked to maintain their current life habits. Measurements were carried out before and after intervention, and in addition, some variables were also measured at the three-month time point.

A target number of 150 healthy participants have been continuously recruited. All healthy participants in the initial contact have to pass the same evaluations as their sleep disordered counterparts. After the baseline assessments, a subgroup of the healthy participants are asked to participate in the same extensive sleep measurements and follow-up tests as the apnea group in order to serve as a reference group (target n = 12).

**Figure 1:**
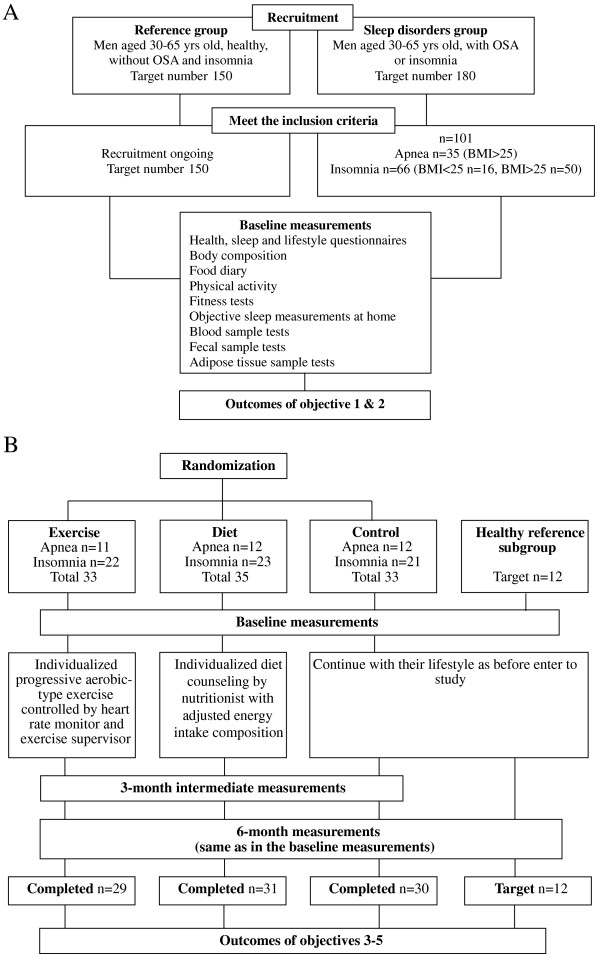
**Flow diagram of the study design. (A) **study design summary of objectives 1 and 2; **(B)** study design summary of objectives 3 to 5.

The trial is registered under http://www.controlled-trials.com: ISRCTN77172005. A summary of the study design is presented in Figure 1.

### Hypotheses

    1. Physical activity is beneficial in terms of good quality and proper duration of sleep. The levels of melatonin, cortisol, and neuro-active steroids are modified by physical activity and thereby influence sleep.

2. The amount of micronutrients in the diet, such as tryptophan and the vitamin Bs, are correlated with quality and duration of sleep.

3. Six-month guided individualized exercise or diet counseling intervention can independently alleviate sleep disorders among adult men with sleep disorders. The improvement can be observed by changes in duration of total sleep, duration and proportion of slow-wave sleep (SWS) and rapid eye movement (REM) sleep, stress reactions based on heart rate variability (HRV) and AHI (participants with OSA), as well as subjective sleep measures such as the Epworth Sleepiness Scale score.

4. Prolonged good quality of sleep after six-month individualized exercise intervention is linked to decreased oxidative stress that in turn may affect melatonin levels in the peripheral circulation because indole is rapidly used to combat free radical damage. Exercise also affects cortisol and neuro-active steroid levels. The mutual interactions between exercise and these hormonal milieus are responsible for sleep-wake cycle regulation.

5. Six-month guided individualized exercise and diet counseling intervention can improve sleep quality through modification of gut microbiota composition. The composition of the gut microbial community is host-specific, evolving throughout an individual’s lifetime and susceptible to both exogenous and endogenous modifications. Hence, the cross-talk between gut hormones and hypothalamic factors (gut-brain axis) is important in the regulation of food intake and sleep disorders.

### Primary and secondary outcomes

#### Primary endpoints

Duration and proportion of sleep stages, heart rate, heart rate variability, respiration and body movements during sleep, and AHI (PSG for participants with OSA;pressure sensor measurement for both OSA and other participants)

Subjective measures of duration and quality of sleep (Nordic sleep disorder questionnaire, Epworth Sleepiness Scale, seven-day sleep diary)

Lifestyle risk clusters related to sleep disorders (healthy versus sleep disordered patients)

#### Secondary endpoints

Anthropometry (body weight and height; waist, hip, chest and neck circumferences)

Blood pressure

Body composition (dual-energy x-ray absorptiometry and bioimpedance)

Fitness (2 km walking test, three-minute step test)

Composition of gut microbes (fecal sample)

Subcutaneous adipose tissue metabolism (total RNA)

Venous blood samples (total cholesterol, HDL-cholesterol, LDL-cholesterol, triglycerides, glucose, insulin, histamine, tryptophan, melatonin, cortisol, adrenalin, leptin, adiponectin, neuro-active steroids, fatty acids, B vitamins, fatty acid profile, and inflammatory variables such as CRP, IL-6, TNF-alpha, and so on)

### Participants

Participants in this study consist of a target sample of 330 Finnish men aged 30 to 65 years.

Participants with sleep disorders: a target number of 180 men with sleep disorders are to be randomized into exercise or diet intervention groups. After screening and physician examination, 35 men with diagnosed mild to moderate OSA and 66 with insomnia confirmed by a physician have been included.

Inclusion criteria for apnea and insomnia patient groups:

• Men in an age range of 30 to 65 years

• Occasionally physically active or sedentary (regular leisure-time exercise ≤ two times per week and ≤ 45 minutes per session)

• Report recurrent difficulty in falling asleep, too short sleep duration or poor quality of sleep during past two months, or with mild to moderate OSA (AHI 5 to 30/hour) with or without continuous positive airway pressure (CPAP) treatment. If under treatment, nasal CPAP for a minimum period of six months prior to the baseline measurements, with a minimum adherence to CPAP therapy for four hours per night

• Relatively healthy (free from cardiovascular comorbidities)

Exclusion criteria:

• Diseases and medications related to: insulin dependent diabetes mellitus, Crohn’s disease, sarcoidosis, celiac disease, thyroid, liver and severe heart diseases, chronic diarrhea, ulcerative colitis, rheumatoid arthritis, severe osteoarthritis, systemic lupus erythematosus, and cancer during the past three years

• Currently taking a special diet (such as a very low calorie diet)

• Other diagnosed sleep disorders (such as narcolepsy)

• Shift workers (working during the night)

• Reported cognitive impairment

• Using antibiotics during the previous three months

• History of eating disorders

• Participants using CPAP, professional drivers and other professions that are at risk of accidents due to discontinuation in CPAP treatment

• Not suitable for the study by a physician’s evaluation

Healthy participants: a target number of 150 healthy men without sleep disorders are intended to be recruited as the reference group. Sixty-three overweight/obese (BMI from >25 to <38) and 44 normal body weight (BMI <25) men with no diseases and taking no medications have been included so far.

Inclusion criteria for reference (healthy men) group:

• Men in an age range of 30 to 65 years

• No chronic sleep disorders and taking no medications related to sleep disorders

• No disease and medications during past one year

• Relatively healthy (free from cardiovascular comorbidities)

Exclusion criteria for reference (healthy men) group are the same as for the patient group.

### Randomization

The apnea and insomnia patients are randomized into the following groups using case-matched computer-generated random number according the participants enrollment order:

Exercise intervention group, n = 33 (apnea = 11 and insomnia = 22)

Diet intervention group, n = 35 (apnea = 12 and insomnia = 23)

Control group, n = 33 (apnea = 12 and insomnia = 21).

### Exercise intervention

In this study, exercises are selected as a combination of Nordic walking, stretching, strength, and relaxation. An individual progressive exercise program, based on the fitness test result at the baseline, is set up in a wrist computer (M5, Suunto Oy, Valimontie 7, 01510 Vantaa, Finland). The exercise is supervised by a specialized trainer once a week and the participants follow the guided exercise program three to five times per week, 30 to 60 minutes per session, at the level of 60 to 75% of the maximum heart rate. The participants transfer their exercise data to the study server via the internet. The participants’ performance is checked and their exercise program updated once a month by the trainer. Each participant has an individual account which is only accessible by him and by the coordinator and researchers of the study. The guided individualized exercise training program is planned to last for six months (26 weeks).

### Diet intervention

Specific individualized diet programs are developed after baseline assessments of each participant’s current dietary intakes (based on three-day food diary) and body weight. Dietary suggestions are given to each individual through group and individual face-to-face counseling during the intervention. The suggested diet contains energy of 40% carbohydrate with < 5% sucrose, 40% fat (saturated fatty acid (SAFA) 10%, monounsaturated fatty acid (MUFA) 15– to 20%, polyunsaturated fatty acid (PUFA) 10%) and 20% protein. In addition, a rich source of vitamins and other micronutrients such as calcium, magnesium, potassium, folate, pyridoxine, cobalamin and choline is also recommended in the guidance. Overweight/obese participants are advised to moderately reduce their total energy intake (by 300 to 500 kcal per day for the first three months) with guidance on the proportion of macronutrients to be consumed. The target is to reduce body weight by 3 kg in the first three months of the intervention. After this period, the participants are advised to maintain their achieved body weight reduction, and continue to gradually reduce their body weight towards normal levels, with a target of a 10% reduction from their initial body weight. Participants with normal weight are advised to maintain their body weight.

Diet intervention is controlled by various methods. An online nutritional counseling service (MealTracker) is used in this study. Participants are asked to send photos of all food intakes during one day to the service 1 to 2 days a week (randomly selected by researcher). The photos are sent by cellphone (mobile application) or computer (MealTracker website, http://www2.mealtracker.fi). The study nutritionist analyzes dietary composition through the photos uploaded and gives feedback via text messages or E-mail to each participant weekly during the first month, and monthly during the rest of intervention. Each participant has an individual account on the server which was only accessible by himself, nutritionists, and researchers of the study. Food diary information is also collected at the three-month time point in order to give more dietary suggestions. Moreover, there are two opportunities for each participant in the dietary intervention group to attend cooking lessons. The cooking lessons are held during the first and second three months of the intervention, respectively. Each time, five or six participants are taught by the nutritionist to cook food that meets the nutritional criteria of this study, and to exchange dietary information.

### Control group

The control group members are asked to maintain their normal activity and diet during the intervention. After the intervention has finished, they are provided with the opportunity of taking three months simultaneous exercise and dietary counseling following the same protocol as the intervention groups.

### Study examinations

All the measurements are carried out before and after six months intervention at the Laboratory of Sport and Health Sciences, University of Jyväskylä. In addition, sleep questionnaire and fitness measurements are also carried out at three months. The measurements are listed below.

Background information regarding lifestyle as well as medical history is collected by questionnaire. Data gathered from eligible participants is used to describe the study populations. Data on self-evaluation of sleep disorders is collected by using the Nordic sleep disorder questionnaire, Epworth Sleepiness Scale as well as a sleep diary [46,47]. Daily physical activities are recorded before, during and after intervention up to seven days for type and duration of all physical activities.

A three-day food diary (two working days and one weekend day) is recorded by all participants. The diary includes type and estimated amount of all food and drink intake during each day. The food records contain time of eating, items and portion of food. Details of all foodstuffs, dishes and drinks including the type and commercial brand name were filled in the records. All the food consumption data are coded into a nutrition program. The mean daily food consumption by main groups and the energy and nutrient intakes are calculated using a Micro-Nutrica software PCprogram developed and maintained by the Research Center of the Social Insurance Institution, Finland (Nordenskiöldinkatu 12, 00250 Helsinki, Finland) [48]. The Micro-Nutrica database contains 66 dietary factors, 680 different food items, and about 640 dishes commonly consumed in Finland.

Health condition examination: a physician examines the physical condition of participants and checks their health history and medications to ensure they meet the inclusion criteria for the study.

Anthropometry and body composition assessments:height and weight are measured and used to determine body mass index (BMI, weight(kg)/height^2^(m)). Chest, waist, hip and neck circumference are measured by using a measuring tape in the conventional way. Blood pressure is measured after five minutes rest. Lean mass, fat mass and bone mass of the whole body are assessed by using dual-energy X-ray densitometry (DXA Prodigy, GE Lunar, OH, USA).

Venous blood samples are taken in standardized fasting conditions in the morning between 7 and 9 am. Serum samples are kept frozen at −80°C until assayed. Biochemical assessments include total cholesterol, HDL-cholesterol, LDL-cholesterol, triglycerides, glucose, insulin, histamine, tryptophan, melatonin, cortisol, adrenalin, leptin, adiponectin, neuro-active steroids, fatty acids, B vitamins and hormones.

Fecal samples are collected by participants at home with tools provided by the study group, under detailed instruction. The samples are stored at −20°C after collection until assayed. The composition of microbiota is measured by using 16S rRNA-hybridization and DNA-staining.

Subcutaneous adipose tissue samples are taken from a subgroup at the waist by a physician. Total RNA is extracted by using the Trizol method (Life Technologies Corporation, 5791 Van Allen Way, Carlsbad, CA 92008, USA). Reverse transcription reaction followed by real-time PCR with gene-specific primer sequences is carried out.

Fitness test: two types of fitness tests are performed: UKK 2 km Walk Test and YMCA Step Test, with a one-hour rest between the two tests. The UKK Walk Test is performed by walking two kilometers as fast as possible on a flat surface [49]. The result is recorded as a fitness index. This index is used to determine the individualized exercise program for those in the exercise group. Both tests are safe for obese people and well represent their fitness level.

Polysomnography (PSG measurements including, for example, EEG, EOG, EMG, movement, ECG and transcutaneous carbon dioxide) is used to evaluate sleep stages and quality, including the non-rapid eye movement (NREM) and rapid eye movement (REM) sleep in all OSA participants in their homes. In addition, seven-night sleep measurements both before and after intervention are taken for all participants by using a non-contact sleep monitoring system at their homes (Beddit sleep tracker, Beddit.com Oy, Kimmeltie 3, 02110 Espoo, Finland) [50]. The measurement records are sent automatically to the Beddit server via the internet, where sleep analyses including sleep stages, HRV and stress reactions are carried out simultaneously. The possible conditions which may affect the measurement, such as children and pets in the bedroom are recorded in the sleep diary. A research assistant visits each participant’s home to set up the system before measurements start.

OSA patients using nasal CPAP are required to stop the treatment for seven days before the PSG sleep measurement. After the baseline measurement, all the patients can continue their regular CPAP therapy for the next six months. After six months of nasal CPAP therapy and intervention, all the patients again stop using their CPAP for seven days before the follow-up PSG measurement. Stopping using CPAP treatment for seven days has no risk for mild and moderate OSA patients, and this is ensured by the physicians who have sufficient experience of OSA treatments and research. Moreover, if the participant experiences intense tiredness due to the discontinuation of CPAP, the PSG measurement is arranged after a shorter discontinuation period.

## Discussion

Currently there is a lack of evidence regarding the associations between lifestyle, metabolism, and sleep. This study focuses on middle-aged men with two different sleep disorders; OSA and insomnia. Through a six-month intervention with either aerobic exercise or optimized diet, results from a range of sleep assessments will provide a clear information about differences in sleep duration, sleep stages, and quality of sleep among the different groups. A comparison between subjective sleep evaluation and objective sleep measurement results among sleep disordered men will also be possible with the data from this study. Variables in the physiological dimension are important references for explaining mechanisms behind sleep and lifestyle. Measurements of neurotransmitters, gut microbiota composition, and adipose tissue characteristics before and after intervention in this trial may strengthen evidence for links between lipid metabolism and sleep outcomes, or find new associations in related fields.

Comparing the participants who have sleep disorders to their healthy counterparts will allow us to find out which lifestyle risk factors are associated with sleep quality and duration. The detailed information collected in this study regarding levels of physical activity and different composition of food intakes and micronutrients will be used to test our hypotheses 1 and 2. This information can be used for diagnosing risk factors and developing healthcare guidance related to sleep disorders.

In this study we applied modern technology combined with socio-psychological support and self-commitment to ensure the participants’ compliance during the intervention. The online exercise diary service and remote food analysis and counseling have been rarely, if ever, used in previous studies and thus may enhance the efficacy and reliability of the intervention. We believe that allowing participants to monitor their progress more effectively could be a potent tool in helping them to change inactive and unhealthy lifestyles.

The current burden of sleep disorders is associated with sedentary lifestyles and unhealthy diets [37,51]. In Finland, great effort has been made to use exercise and dietary intervention to prevent chronic disease clusters and reduce the related socio-economic burden, such as in the context of cardiovascular disease [52]. This study is expected to provide abundant experimental and cross-sectional results, which may shed light on many aspects of sleep and metabolism and the interaction between the two.

### Ethical and data protection issues

The study is approved by the Ethic Committee of the Central Finland Health Care District (7/2011 OTE). Participants in this study are volunteers. None of the measurements are known to entail any significant health risk. The study has its own physician to ensure the eligibility and safety of participants. All data are handled and archived confidentially and are registered with the Finnish National Data Protection Ombudsman. The benefits and associated risks of the study are carefully explained and the voluntary nature of the participation is emphasized. Informed consent is obtained from all participants prior the baseline measurements. If the participant agreed to participate, a copy of the signed consent form is kept in his records.

### Trial status

Participant recruitment for intervention started in April 2011. Baseline measurements were taken between June and December 2011, and all six-month exercise and diet interventions with follow-up measurements for the intervention groups were completed by July 2012. Participants in the control group who attended the extra three-month exercise plus diet intervention finished the follow-up measurements by January 2013. Feedback meetings for participants have been held twice to explain the preliminary results of their health and sleep quality after the completion of intervention. Biomarker assays of the study are continuously under analyses. The recruitment of the healthy reference group started from June 2012 and will be completed by April 2013. Feedback for the healthy participants is planned to be given during June and July 2013.

## Abbreviations

AHI: apnea hypopnea index; BCG: ballistocardiography; BMI: body mass index; CPAP: continuous positive airway pressure; CRP: C-reactive protein; CVD: cardiovascular disease; ECG: electrocardiography; HPA axis: hypothalamic-pituitary-adrenal axis; HRV: heart rate variability; IL-6: interleukin-6; MUFA: monounsaturated fatty acid; NREM: non-rapid eye movement; OSA: obstructive sleep apnea; PCR: polymer chain reaction; PSG: polysomnography; PUFA: polyunsaturated fatty acid; REM: rapid eye movement; SAFA: saturated fatty acid; SWS: slow-wave sleep; TNF-alpha: tumor necrosis factor-alpha.

## Competing interests

The authors declare that they have no competing interests.

## Authors’ contributions

CS is the principle investigator of the study and has full access to all of the data in the study and takes full responsibility for the integrity of the data and for the accuracy of the data analysis. None of the authors have financial or personal interest affiliations with the sponsors of this research effort. Study concept and design: CS, TX, MTM, PM and AM. Acquisition of data: TX, SA, MTM, TJ, MS, RA, CSM, PS, WP, ME, WXF, AM and CS. Analysis and interpretation of data: TX, SA, MTM, CSM, EN, TJ, MS, PS, WP, ME, WXF, CFY, WX, ZYJ, TI, SYN, PM, AM and CS. Drafting of the manuscript: TX, MTM and CS. Critical revision of the manuscript for important intellectual content: TX, SA, MTM, CSM, TJ, MS, RA, EN, PS, WP, ME, WXF, CFY, WX, ZYJ, TI, SYN, PM, AM and CS. Funding obtaining: CS, SYN and AM. Administrative, technical, or material support: CSM, MTM, TJ, MS, RA, PS, SYN, AM and CS. Supervision: CS, CFY, TI, SYN and AM. All authors read and approved the final manuscript.
